# Highly Active W_2_C-Based Composites for the HER in Alkaline Solution: the Role of Surface Oxide Species

**DOI:** 10.1021/acsami.4c01612

**Published:** 2024-04-22

**Authors:** S. Díaz-Coello, D. Winkler, C. Griesser, T. Moser, J.L. Rodríguez, J. Kunze-Liebhäuser, G. García, E. Pastor

**Affiliations:** † Institute of Materials and Nanotechnology, Department of Chemistry, University of La Laguna, PO Box 456, 38200 La Laguna, Santa Cruz de Tenerife, Spain; ‡ Department of Physical Chemistry, University of Innsbruck, Innrain 52c, Innsbruck 6020, Austria

**Keywords:** HER, tungsten carbide, suboxide, ionic liquid, AEMWE

## Abstract

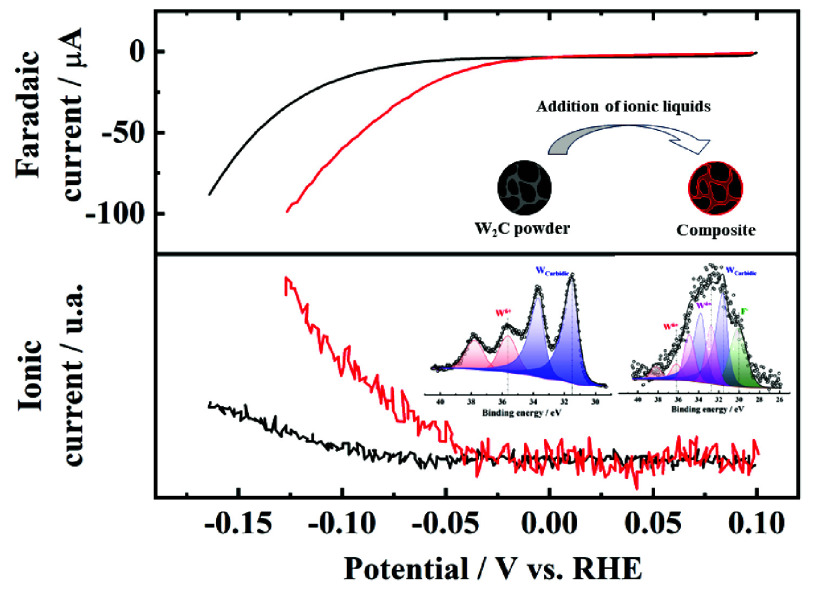

The hydrogen evolution reaction (HER) is a crucial electrochemical process for the proposed hydrogen economy since it has the potential to provide pure hydrogen for fuel cells. Nowadays, hydrogen electroproduction is considerably expensive, so promoting the development of new non-noble catalysts for the cathode of alkaline electrolyzers appears as a suitable way to reduce the costs of this technology. In this sense, a series of tungsten-based carbide materials have been synthesized by the urea-glass route as candidates to improve the HER in alkaline media. Moreover, two different pyridinium-based ionic liquids were employed to modify the surface of the carbide grains and control the amount and nature of their surface species. The main results indicate that the catalyst surface composition is modified in the hybrid materials, which are then distinguished by the appearance of tungsten suboxide structures. This implies the action of ionic liquids as reducing agents. Consequently, differential electrochemical mass spectrometry (DEMS) is used to precisely determine the onset potentials and rate-determining steps (RDS) for the HER in alkaline media. Remarkably, the modified surfaces show high catalytic performance (overpotentials between 45 and 60 mV) and RDS changes from Heyrovsky–Volmer to Heyrovsky as the surface oxide structures get reduced. H_2_O molecule reduction is then faster at tungsten suboxide, which allows the formation of the adsorbed hydrogen at the surface, boosting the catalytic activity and the kinetics of the alkaline HER.

## 1 Introduction

As environmental issues increase, the obtention of better and cheaper methods for the generation of high-purity green hydrogen is rising. In this context, low-temperature water electrolysis (WE) technology is one of the most promising green methods to overcome this necessity.^1^ Nowadays, several types of WE technologies are available. Proton exchange membrane water electrolyzers (PEMWE) and alkaline water electrolyzers (AWE) are two important examples, but they still present some inherent problems that need to be solved.

First, AWE's strength lies in the use of low-cost cathodic catalysts. These generally consist of Ni-based materials operating in high-concentration alkaline solutions (6–9 M KOH) at temperatures between 60 and 90 °C.^2^ AWE technology is industrially mature and well established, but its performance can only reach low working current densities (0.2–0.4 A cm^–2^) at low operating pressure (<30 bar).^3^ These disadvantages arise from the experimental configuration of the electrolyzers, which implies the use of a diaphragm assembly for the conduction of hydroxide anions. Originally, asbestos was the material used for the construction of AWE diaphragms as it could resist the strong alkaline environment.^4^ Thus, very poor current–voltage responses due to high ohmic resistance produced by the separator forced the development of new diaphragm materials in the last decades. Some examples are the Zirfon^5^ and, more recently, the development of thin film composites^6^ or thermoplastic polymer-based materials.^7^ However, the development of diaphragm materials and the improvement of electrode coatings and cell configuration are not sufficient to overcome some of the inherent drawbacks of this technology. Indeed, the possibility of gas crossover through the diaphragm along with the formation of insoluble carbonates (by contact of the solution with atmospheric CO_2_) is a major problem that still need to be solved.^8^

In this sense, the use of proton exchange membranes in PEMWE technology solves all assembly issues caused by the diaphragm and the high-concentration alkaline solution in AWE devices. PEM configuration implements weakly acidic electrolytes allowing low ohmic losses. Accordingly, high operational current densities can be reached (0.6–2.0 mA cm^–2^) along with higher hydrogen pressures than AWE technology (<200 bar).^3^ An issue of PEM devices is the use of an acid-activated polymer with high proton conduction (e.g., Nafion) as membrane, where local acidic conditions can occur^9^ that lead to local pH decreases. Therefore, the use of corrosion-resistant HER catalysts is required. So far, the catalysts that can operate under these conditions usually belong to the platinum group metal (PGM) for both cathode and anode.^10^ Since PGMs are much more expensive than the catalysts needed for AWE, the cost of hydrogen increases from ∼1000 to ∼2000 € kW_el_^–1^ for PEM electrolyzers.^3^

This being considered, Anion Exchange Membrane Water Electrolysis (AEMWE) appears as a technology developed to exploit both AWE and PEMWE advantages.^10,11^ This merged technology includes a polymer membrane-based assembly electrolysis device running in an alkaline solution, as traditional water electrolyzers do. Therefore, this configuration should allow us to (i) use non-noble catalysts, as the environment is less corrosive than acidic medium; and (ii) reduce the concentration of electrolyte similarly to PEMWE technology and thus avoid carbonation issues. However, AEMWE is still under development, both in terms of finding new low-temperature anion exchange membranes and in the implementation of new low-cost catalysts with high catalytic performances (both cathodic and anodic).^12,13^

Ni-based catalysts are the most studied materials for use in AEMWE technology as they already exhibit acceptable electrocatalytic performance. However, those catalysts often undergo fast deactivation when cathodic potentials are applied. To overcome this problem, some catalysts based on Ni–Fe alloys,^14,15^ Cu modified with Ni-oxides,^16,17^ transition metal dichalcogenides,^18^ and transition metal nitrides^13^ have been recently reported. Transition metal carbides (TMCs) have also revealed acceptable catalytic activities toward the hydrogen evolution reaction (HER) in an alkaline solution.^19^ In this sense, it is well-known that the electronic structure of several carbides (especially tungsten-based ones) presents some theoretical similarities to the electronic structure of platinum.^20^ Since this was first proposed, several articles reported reasonable catalytic activities of carbide-based toward the HER.^20−22^ Nevertheless, carbides usually present complex surface chemistry changes due to oxidation processes that lead to the formation of the corresponding transition metal oxides (TMOs).^23−25^ These surface oxides are reported to play a central role in the electrocatalytic behavior of the material toward several reactions.^25,26^

On the other hand, ionic liquids (ILs) have raised expectations in electrochemistry due to their features^27−29^ as they are tunable in terms of their specific properties (e.g., melting point, hydrophobicity, solubility, etc.). The concept of solid catalysts with ionic liquid layers is known from the supported ionic liquid phases (SILPs) previously applied for heterogeneous catalysis in the solid/gas interface.^30,31^ Based on that idea, some authors have tried to apply the same type of modification to electrochemical catalysts in aqueous media. In this sense, some interesting works have been reported by Erlebacher et al. for oxygen reduction catalysts using an ionic liquid as a modifier. They modified NiPt surfaces^32^ and carbon-supported Ni/Pt encapsulated nanoparticles.^33^ As the main conclusions on solid catalysts with ILs for aqueous experiments, the authors conclude that the latter must have (i) protic structures allowing high proton conduction; (ii) high hydrophobicity; and (iii) a good mechanical resistance for the immobilization of the IL onto the surface or within the catalyst pores. Apart from these studies, we have also reported the improvement of HER electroactivity using composite materials generated with commercial TMCs and ILs.^34,35^ In those cases, pyridinium-based ILs were selected as they present all of the properties mentioned above and provide high electrical and ionic conductivities. Indeed, the addition of the selected ILs to commercial W_2_C, WC, and Mo_2_C powders showed a synergistic effect during the HER in alkaline solution.^34,35^

A central parameter to understanding and improving the performance of electrocatalysts is the rate-determining step (RDS) of the reaction. Regarding hydrogen generation through water splitting in alkaline media, the widely accepted mechanism involves three possible steps:
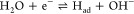
1
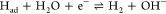
2
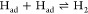
3

The elucidation of the RDS is related to the HER Tafel slopes, as previously reported.^36^ A Tafel slope of 120 mV dec^–1^ implies Volmer reaction 1 as the RDS, a slope of 40 mV dec^–1^ suggests Heyrovsky reaction 2 as the RDS, and a slope of 30 mV dec^–1^ infers Tafel reaction 3 as the slowest step for the whole process. In alkaline solution, the process begins with the dissociation of a water molecule which severely affects the rate of the Volmer step. Markovic et al.^37,38^ proposed that the water splitting in an alkaline solution is controlled by the adsorption energy of the OH and the energy of the H–OH bond of the water molecule in addition to the adsorption energy of H. It was shown that the addition of Ni(OH)_2_ to platinum surfaces leads to an enhancement of HER activity due to the interaction of the water molecule with both surfaces. This interaction is considered responsible for a decrease of the energy needed to break the H–OH bond producing the adsorption of hydrogen onto the catalytic surface. Thus, the presence of transition metal hydroxides can lead to an increase in the activity of the surface toward the alkaline HER on both Pt(111)^37,38^ and Cu(111).^17^

The aim of this work is to explain the influence of pyridinium-based ILs on the formation of composite materials with tungsten-based carbide nanoparticles from a surface chemistry and kinetic point of view. In this sense, two W_2_C powders with different surface carbide/oxide ratios have been synthesized and modified with ionic liquids. X-ray photoelectron spectroscopy (XPS) and Raman spectroscopy have been employed for the physicochemical characterization of the surfaces. The HER onset overpotentials are determined with differential electrochemical mass spectrometry (DEMS). To unravel the RDS of the catalysts prepared in this work, Tafel slopes have been calculated from the currents of the *m*/*z* = 2 signals recorded with DEMS. This allows a precise determination of this parameter as it only contemplates the contribution associated with the electrochemically generated hydrogen without including side reactions (e.g., surface oxide reduction currents).^39^

## 2 Experimental Methods

### 2.1 Synthesis of Tungsten-Based Materials

Tungsten-based nanoparticles have been synthesized by a slight modification of the urea-glass route, first proposed by Giordano et al.^40,41^ Briefly, 1 g of WCl_4_ (Sigma-Aldrich, 99.9%) was mixed with 2.6 mL of dehydrated ethanol (Merck, emsure) under continuous stirring conditions in an argon atmosphere (99.999%, Air Liquide) for 30 min. Then dried urea (Sigma-Aldrich, p.a.) was added to the solution under stirring and argon atmosphere for 3 h. After that, the precursor was divided into two crucibles and two samples were obtained by thermal treatments in a tubular furnace (Carbolite, UK) from room temperature to 800 °C with different heating rates (2 and 4 °C min^–1^ for the catalysts denoted as WCU2 and WCU4, respectively) under argon flux (130 mL min^–1^). Commercial W_2_C was purchased from Alfa Aesar and employed for comparison purposes.

### 2.2 Catalyst Ink Preparation

The synthesis and characterization of ethylpyridinium hexafluorophosphate (EPy) and octylpyridinium hexafluorophosphate (OPy) was recently reported in previous studies.^34,35^ Catalyst inks were prepared in the absence (i) and presence (ii) of EPy or OPy by ultrasound-assisted physical mixing as follows:

(i) 20 mg of tungsten-based material (WCU2, WCU4, or commercial W_2_C) + 15 μL of Nafion solution (5 wt %; Aldrich) + 500 μL of isopropanol (Merck, p.a.).

(ii) 19 mg of WCU4 + 1 mg of ionic liquid (EPy or OPy) + 15 μL of Nafion solution + 500 μL of isopropanol. The obtained composite materials are labeled in this work as WCU4-EPy and WCU4-OPy.

### 2.3 Physicochemical Characterization

X-ray diffraction (XRD) patterns were detected using a universal diffractometer PANanalytical X’Pert Pro operating with Cu Kα radiation (λ = 0.1550 nm) generated at 40 kV and 20 mA. The scan rate of the instrument was set at 0.04 grade s^–1^ for 2θ values between 20° and 85°. Apparent crystallite sizes were achieved from the Scherrer equation. Rietveld analysis and crystalline composition were reached with MAUD software (Material Analysis Using Diffraction) and the Inorganic Crystal Structure Database (ICSD).

Raman spectra were recorded by using a SPELEC RAMAN instrument (Metrohm DropSens) equipped with a λ = 532 nm laser. Scanning electron microscopy (SEM) pictures have been achieved using a Tescan Clara microscope operating at 30 kV with a secondary electron detector. Images have been obtained for all of the materials studied in this work.

X-ray photoelectron spectroscopy (XPS) measurements were carried out with Multilab 2000 equipment and an Alpha 110 hemispherical sector analyzer (Thermo Fisher Scientific). The X-ray source used monochromated Al Kα (1486.6 eV) radiation. Charge compensation was performed using a flood gun (emission of electrons at 6 eV of kinetic energy) and charge-induced shifts were corrected with reference to the WO_3_ component (35.8 eV) and with reference to the sp^3^-carbon component at 284.8 eV. Survey scans were recorded at a pass energy of 100 eV and an energy step size of 1 eV while high-resolution spectra were recorded at a pass energy of 25 eV and an energy step size of 0.1 eV for the regions of interest (W 4f, C 1s and O 1s). Deconvolution and semiquantitative analysis of the spectra was performed with CasaXPS software using a Shirley-type background and mixed Gaussian–Lorentzian functions (GL30). An asymmetric peak shape was used to fit signals of the peaks related to W_2_C.

### 2.4 Spectroelectrochemical Characterization

Differential electrochemical mass spectrometry (DEMS) has been used to elucidate both mechanistic and kinetic parameters of the HER at catalysts employed in the current work. DEMS measurements were performed using a three-electrode half-cell (Hiden Analytical) at room temperature controlled by an AutoLab PGSTAT302 potentiostat–galvanostat (Metrohm Autolab). A hydrophobic PTFE membrane (pore size = 0.2 μm, Cobetter filtration) was placed at the interface between the cell and the mass spectrometer chamber, and the working electrode was employed in a thin layer configuration. Mass-to-charge ratio (*m*/*z*) = 2 was set to follow the production of hydrogen using a secondary electron multiplier detector.

A carbon rod was used as a counter electrode, while the reference electrode was a KCl-saturated silver/silver chloride electrode and the supporting electrolyte was 0.1 M NaOH (Merck, p.a.). All potential values in this work have been converted and referred to the reversible hydrogen electrode (RHE). The working electrode consisted of 20 μL of the respective catalyst ink deposited onto a glassy carbon (GC) disc (geometric area: 0.3848 cm^2^) and dried under Ar atmosphere. Then, the working electrode was introduced into the cell at a controlled potential of 0.1 V and the potential was swept toward cathodic values to record simultaneously linear sweep voltammograms (LSVs) and mass linear sweep voltammograms (MSLSVs). Further information about the configuration and delay time of the setup can be found elsewhere^25,26^ as well as in Section 3 of the Supporting Information.

## 3 Results and Discussion

### 3.1 Physicochemical Characterization

**Figure 1:**
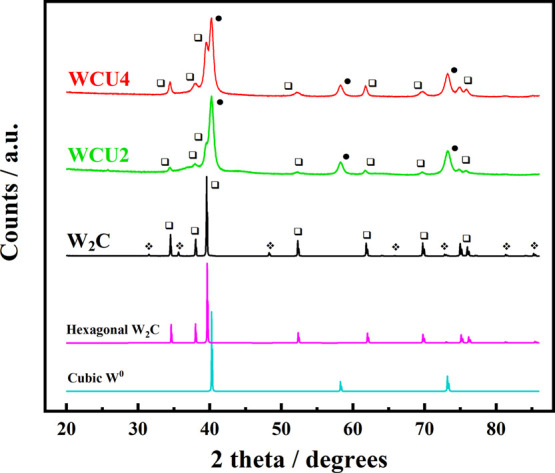
XRD patterns of commercial W_2_C (black line), WCU2 (green line), and WCU4 (red line) materials. Cubic tungsten (•, 00-004-0806; turquoise line) and hexagonal W_2_C (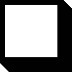, 00-035-0776; magenta) from the PDF2 database are also depicted for comparison purposes. The signals related to WC traces in the commercial carbide are marked with 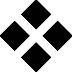.

Figure 1 depicts the X-ray diffractograms for the commercial W_2_C and synthesized materials. The latter shows three intense diffraction peaks located at 2θ = 40.2°, 58.2°, and 73.1°, corresponding to the (110), (200), and (211) crystalline planes of metallic tungsten (W^0^; Im3m; 00-004-0806 PDF2 database), respectively. In addition, commercial W_2_C, WCU2, and WCU4 display diffraction patterns at 2θ = 34.5°, 38.0°, 39.6°, 52.3°, 61.8°, 69.7°, 74.9°, and 75.9° that are associated with the (100), (002), (101), (102), (110), (103), (112) and (201) crystalline phases of hexagonal W_2_C (P-3m1; 00-035-0776 PDF2 database). Noticeably, commercial W_2_C also shows diffraction patterns associated with hexagonal WC (P-62m, 01-073-0471 PDF2 database).

**Figure 2:**
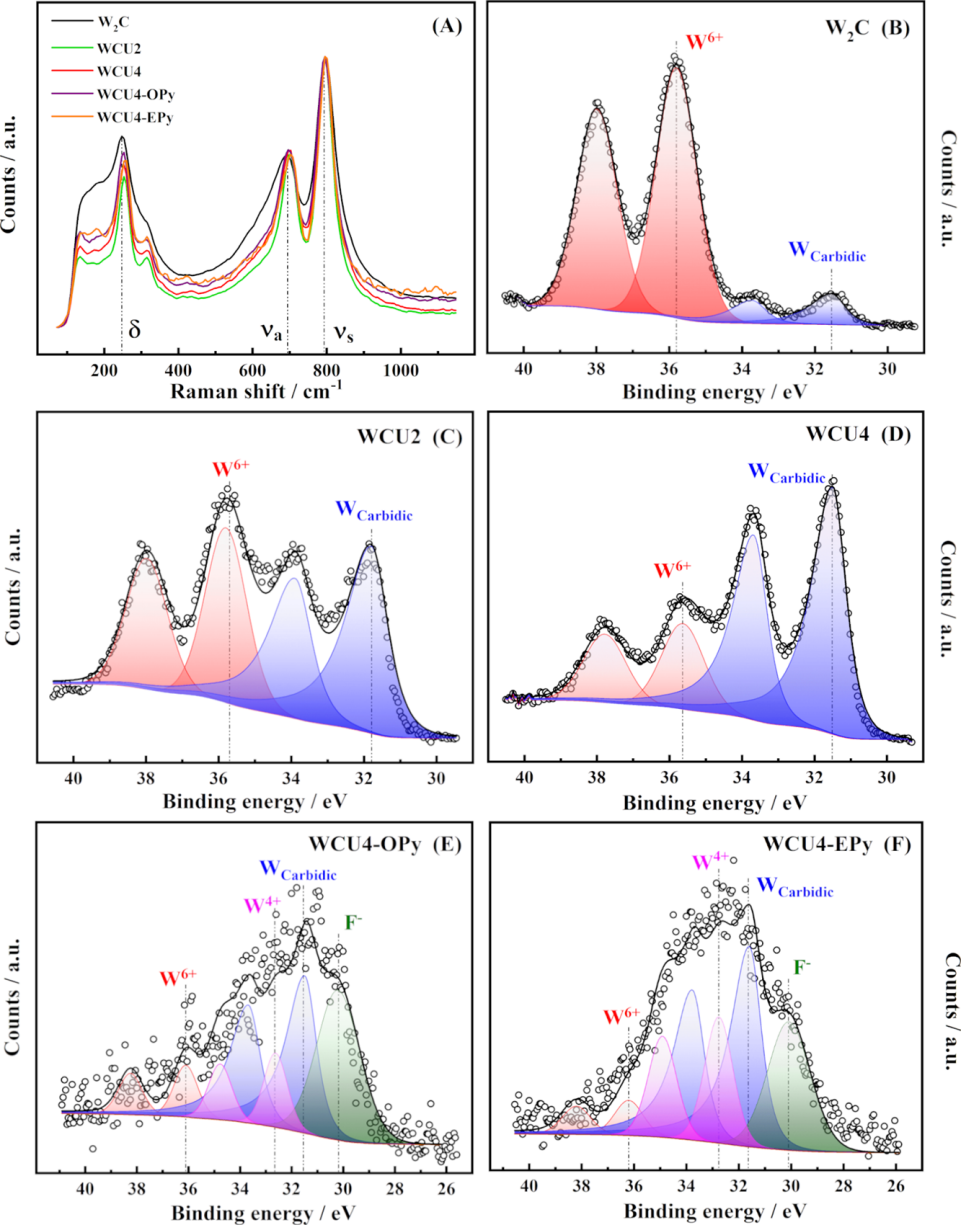
(A) Raman spectra, where δ indicates the O–W–O bending vibrational mode while ν_a_ and ν_s_ indicate the symmetric and antisymmetric W–O stretching mode, respectively. (B–F) High-resolution XPS scans recorded in the W 4f region for W_2_C, WCU2, WCU4, WCU4-OPy and WCU4-EPy. (E,F) show contributions from F 2s in the same region.

X-ray diffractograms and their corresponding Rietveld analysis indicate a higher metallic character of WCU2, i.e., WCU2 and WCU4 present a W^0^/W_2_C ratio of 1.17 and 0.6, respectively. In addition, the Scherrer approximation indicates similar crystallite sizes of crystalline phases for both synthesized materials (W^0^ ∼ 13 nm and W_2_C ∼ 20 nm). This shows that different heating rates produce materials with different compositions of the crystalline phases but with similar crystallite sizes. Commercial W_2_C reveals a crystallite size of 218 nm for the main crystalline phase (W_2_C). Figure 2A depicts the Raman spectra of commercial W_2_C, WCU2, WCU4, WCU4-OPy and WCU4-EPy. All spectra show two strong signals located at ca. 700 and 800 cm^–1^ which have been assigned to the antisymmetric and symmetric stretching of the W–C bond.^42^ On the other hand, the less intense signals located at 260 and 317 cm^–1^ have been assigned to the WC-O bending vibration of surface oxides.^42^ Both crystalline and amorphous oxide structures are reported to have active vibrations in the region between 600 and 950 cm^–1^. In this case, these are expressed as the wide shoulders spotted for the W–C peaks. Therefore, despite the crystalline character of the bulk materials detected by XRD, both synthesized and commercial carbide surfaces comprise amorphous oxide species (which do not appear in Figure 1). Those are most likely formed by reaction with the atmospheric oxygen after the thermal treatment. Indeed, this is a common behavior of carbides in contact with atmospheric air and aqueous electrolytes.^25,43^

Besides Raman spectroscopy, SEM has been used to obtain information about the structure of the powders (see Figure S1). First, there is a clear difference between the structures formed by the commercial W_2_C and the synthesized carbides. In terms of microstructures, the former is distributed in homogeneous grains, while the latter forms heterogeneous structures with several particle sizes. However, a closer inspection reveals the formation of nanostructures on the surface of the latter. This characteristic is present at both WCU2 and WCU4, as well as at the IL-modified catalysts.

XPS analysis of all of the catalysts considered in this work was performed to determine the oxidation states of surface species and their composition. Figure 2B–F shows the high-resolution XPS spectra recorded at the W 4f region for W_2_C, WCU2, WCU4, WCU4-OPy, and WCU4-EPy. A summary of the parameters used for the deconvolutions as well as the surface composition solved for each sample can be found in Table S1, and a complete report of the regions of interest for all studied materials is provided in the Supporting Information (see Figures S2–S6). Figure 2B reveals that commercial W_2_C is protected with a thick oxide film consisting of mainly WO_3_ (W^6+^ signal, 94%), which covers the crystalline W_2_C (spotted by XRD). The synthesized W_2_C powders, WCU2 and WCU4, present a much higher amount of carbide on the surface (Figure 2C,D), achieving a concentration of 46 and 56%, respectively (according to XPS data in Table S1). Thus, WCU2 and WCU4 show a thinner WO_3_ film on their surface, so the synthesis procedure proposed in this work grants the generation of nanoparticles with significant proportions of tungsten carbide available on the surface for the HER to take place. As WCU4 has the lowest oxide coverage on its surface, it was selected for the investigation of the effect of ionic liquid (OPy or EPy) on this material.

We recently reported that OPy reduces the amount of surface oxide and prevents the agglomeration on commercial transition metal carbides, enhancing the catalytic performance toward the HER.^35^Figure 2E,F depict XPS spectra of the W 4f region of WCU4-OPy and WCU4-EPy, respectively. It is interesting to note that WO_*x*_ suboxide (W^x+^) signals appear in these spectra, probably generated as a reduction product of WO_3_ due to the interaction with the ionic liquid. As it was reported in the literature,^44,45^ the formation of tungsten suboxides normally implies the enhancement of electrocatalytic activity toward HER in acidic media. This is caused by the alteration of the electronic structure of the formed suboxide, which promotes a decrease in the change of free energy for hydrogen adsorption (Δ*G*_H_). Later, it will be shown that the formation of tungsten suboxides most likely promotes the formation of H_ad_ in alkaline media as well. Ionic liquids thus act as surface-reducing agents; the reductive strength seems to depend on the length of the aliphatic chain, i.e., the shorter the aliphatic chain, the stronger the reducing agent. An improvement of the catalytic activity toward the HER is therefore anticipated for IL/tungsten carbide composites.

### 3.2 Spectroelectrochemical Characterization toward HER

**Figure 3:**
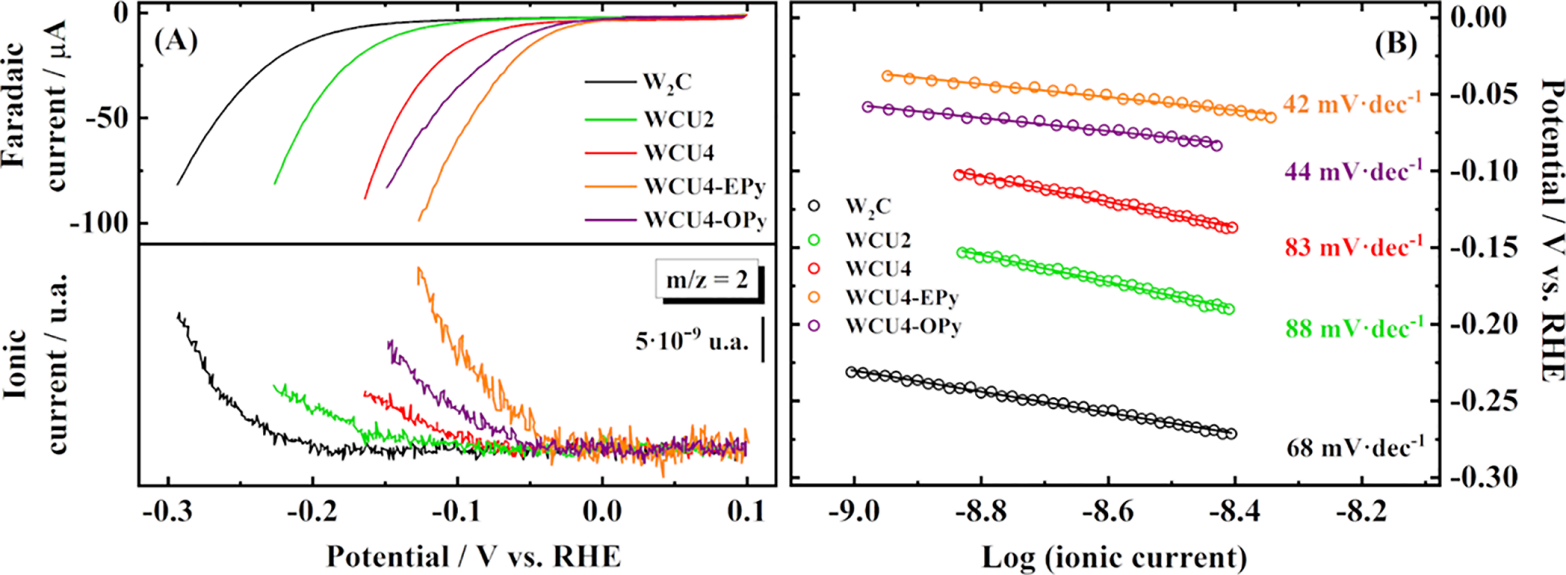
(A) LSVs (top panel) with corresponding MSLSVs for *m*/*z* = 2 (bottom panel) signals obtained in the DEMS experiments performed with W_2_C, WCU2, WCU4, WCU4-OPy and WCU4-EPy catalysts. The LSVs were recorded starting at 0.1 *V*_RHE_ and going toward reduction potentials at 1 mVs^–1^ in 0.1 M NaOH (IUPAC convention) using a GC rod as counter electrode, a KCl_sat_ Ag/AgCl reference electrode and a GC disk as substrate for the working electrode material. The procedure for the modification of the substrate can be found in the experimental section. (B) Tafel plot for the HER obtained from MSLSVs.

DEMS experiments were performed to elucidate kinetic parameters and determine the exact onset potentials of the HER. Figure 3A shows linear sweep voltammetry (LSVs, top panel) and the corresponding mass linear sweep voltammograms (MSLSVs, bottom panel) for the *m*/*z* = 2 signal ([H_2_]^·+^) recorded in the experiments with the W_2_C, WCU2, WCU4, WCU4-OPy and WCU4-Epy catalysts. It is important to note that the *m*/*z* = 2 signal is only associated with the H_2_ formation, and consequently, accurate onset potentials and kinetics parameters for the HER are attained.^39^

**Figure 4:**
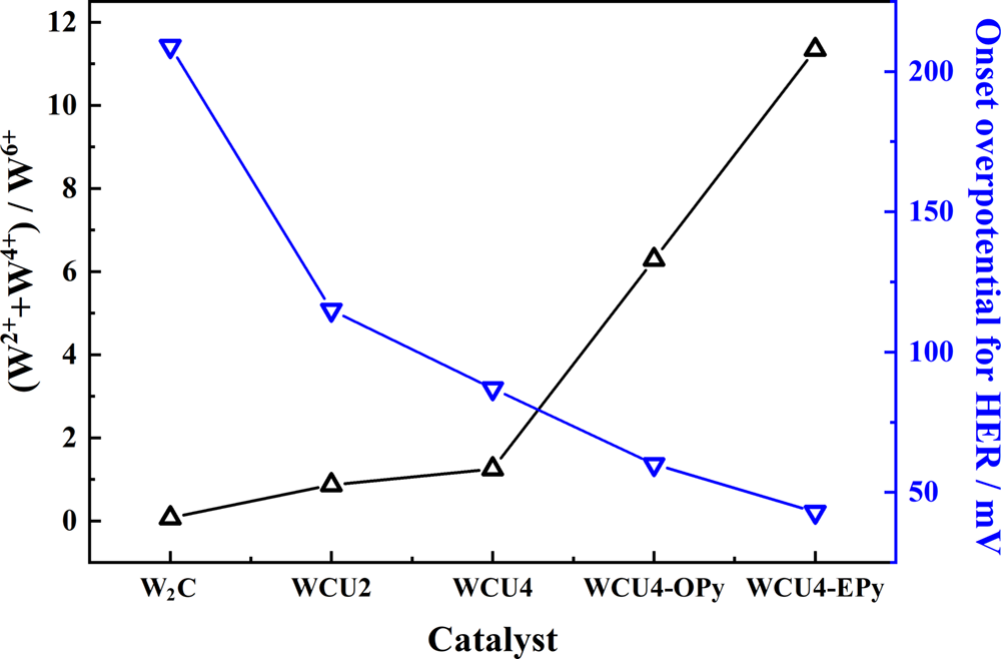
(W^2+^ + W^4+^)/W^6+^ surface ratio (black line and left axis) and onset overpotential for the HER determined by DEMS (blue line and right axis) for all catalysts studied.

In this context, the overpotential for the onset of the HER decreases in the way W_2_C (260 mV) ≫ WCU2 (150 mV) > WCU4 (100 mV) > WCU4-OPy (60 mV) > WCU4-EPy (45 mV). Interestingly, the enhancement of the catalytic activity toward the HER is directly related to the surface composition of the electrode and the presence of suboxides. In this sense, two things are proven. First, for the unmodified carbides, there is a clear trend relating the amount of WO_3_ detected by XPS and the catalytic activity; so, as fewer oxides are detected in the X-ray photoelectron experiment, the catalytic activity increases. Additionally, the overpotential values for the onset of the HER are consistent with other W_2_C-based materials already reported in the literature.^22^ Second, it is also clear that the formation of suboxides is increasing the catalytic activity by 40–55 mV. As evidence, Figure S8 depicts the DEMS measurement for the EPy-modified WCU2 material. The figure shows the same behavior as the IL-modified WCU4, resulting in an enhancement of the catalytic activity toward the alkaline HER. This trend is graphically presented in Figure 4. It is noteworthy that the highest (W^2+^ + W^x+^)/W^6+^ ratio was close to 11.4 in the current work (Table S1) which points that the selected ionic liquids significantly reduce the oxides on the W_2_C, being therefore accessible for H_2_O reduction. Moreover, the specific capacitance (*C*_dl_) of the materials has been solved as an approximation of the electrochemical surface area (ECSA).^46,47^ Thus, several cyclic voltammograms have been recorded for each catalyst at different scan rates in the potential window between 0.05 and 0.3 *V*_RHE_ (Figure S10). The results are consistent with the formation of nanostructures spotted by SEM. In this sense, there is a clear difference between commercial W_2_C (*C*_dl_ = 0.313 mF cm^–2^) and the rest of the synthesized samples (4.471–9.807 mF cm^–2^). However, after the modification of WCU4 with ionic liquids, the specific capacitance slightly decreases. This implies that after the addition of the ionic liquids, the active sites are also decreasing. As the catalytic activity is enhanced for the latter, the effect of boosting the catalytic activity toward alkaline HER is not related to the increase of the ECSA but rather to the formation of the suboxides detected by XPS.

Tafel plots were conceived with the objective of analyzing the kinetic parameters and the operating reaction mechanism of the HER for all of the catalysts studied in this work. Figure 3B displays the Tafel slopes obtained from MSLSVs for each material. Interestingly, ionic currents reveal two different behaviors: (i) W_2_C, WCU2 and WCU4 show Tafel slope values between 68 and 88 mV dec^–1^ indicating a mixed Heyrovsky-Volmer reaction as the rate-determining step (RDS); and (ii) WCU4-OPy and WCU4-EPy depict Tafel slope values of ca. 40 mV dec^–1^ that implies a Heyrovsky reaction alone as the RDS. EIS experiments under HER conditions have also been achieved for all of the catalysts (see Figure S11 and Table S2). The results show a clear trend upon the addition of an ionic liquid to the synthesized carbide, reducing the charge transfer resistance (R_ct_) of the materials as follows: WCU4 > WCU4-OPy > WCU4-EPy (140.0 > 133.4 > 70.2 Ω). These values follow the same trend as the ionic-derived Tafel slopes, indicating that the change in the rate-determining steps also implies the decreasing of the R_ct_ as the Volmer step (electrochemical step) gets more favorable at the surface. Consequently, the reaction mechanism of the HER on W_2_C-based catalysts changes according to the catalyst surface composition. This means that the Volmer step is very slow at WO_3_-rich catalysts but very fast when the oxide is reduced and the suboxides are formed on the surfaces of the particles. Such effect is consistent with the available literature for acidic media but first reported in alkaline media in this work. Therefore, it is proved that in alkaline media the adsorption of hydrogen on the surface of tungsten suboxides is also favored.

**Figure 5:**
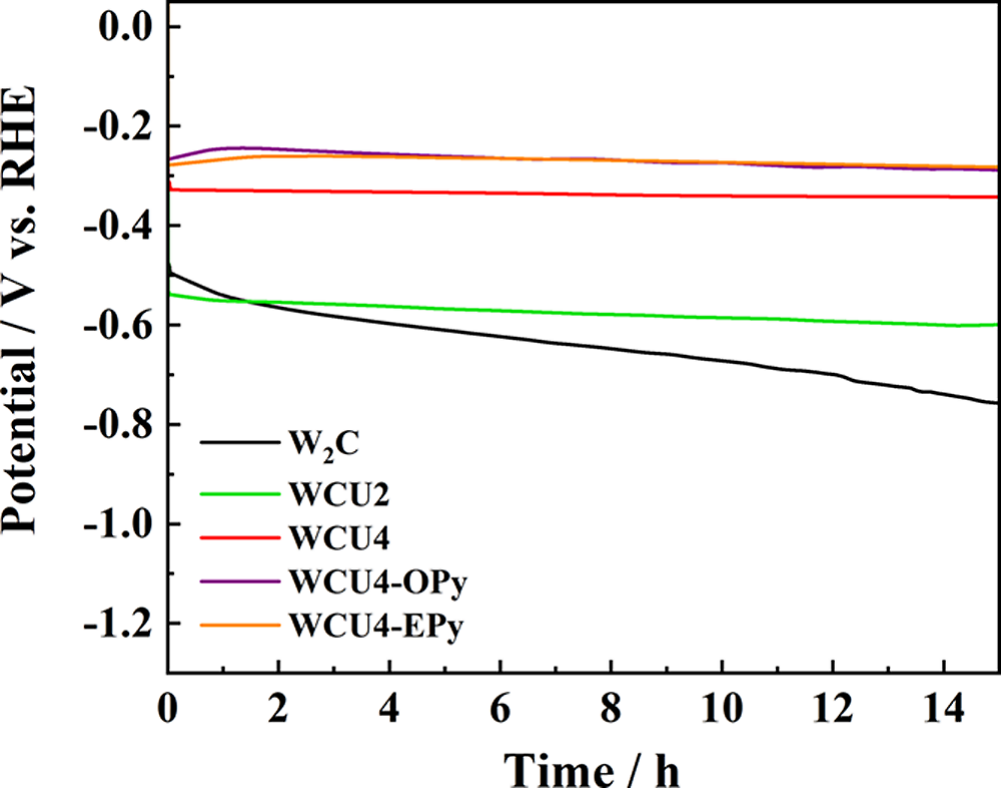
Chronopotentiometry experiments recorded for W_2_C, WCU2, WCU4, WCU4-OPy, and WCU4-EPy catalysts. The catalysts were immersed in the solution under an applied potential of 0.1 *V*_RHE_ for 20 s and then −5 mA cm^–2^ were applied for 15 h in a 0.1 M NaOH solution (IUPAC convention). A GC rod was used as a counter electrode, a KCl_sat_ Ag/AgCl was used as a reference electrode, and a GC disk was used as a substrate for the immobilization of the working electrode material. The procedure for the modification of the substrate can be found in the experimental section.

An important topic about the development of new catalysts lies in studying the stability they present toward the selected reaction. In this sense, several chronopotentiometry experiments were recorded over long periods of time. Figure 5 presents the potential losses for the alkaline hydrogen evolution reaction when a current density of −5 mA cm^–2^ was applied to the catalysts for 15 h. A clear difference arises between the commercial W_2_C, which loses up to 250 mV during the HER; and the synthesized samples, which can be considered stable in the time period considered for the experiment. Therefore, reducing the initial percentage of surface oxide significantly improves the stability of the materials. This is probably caused by the reduction of the high amounts of tungsten oxide detected in the commercial sample by XPS, which is most likely being reduced and dissolved from the surface.^48^ Respecting the IL-modified materials, the increase in catalyst activity proved by DEMS is here corroborated. Thus, while WCU4 operates at approximately −0.33 V vs RHE, the modified catalysts improve their reaction potential under −5 mA cm^–2^ up to −0.26 V vs RHE. However, no special features have been clearly identified about improving the stability of the starting material by mixing it with the ionic liquids. Thus, the losses of potentials are approximately in the same range for the modified and unmodified samples.

## 4 Conclusions

Tungsten carbide catalysts with a crystalline core (consisting of diverse amounts of W_2_C/W^0^) and a surface formed by amorphous WO_3_ and crystalline W_2_C with different proportions were synthesized via the urea-glass route.

The catalyst tagged as WCU4 showed the highest W_2_C/WO_3_ surface ratio, and it was selected to produce composite materials by physically mixing this catalyst with two different ionic liquids (OPy and EPy): WCU4-EPy and WCU4-OPy. Raman and XPS experiments have allowed the surface characterization of all studied materials, showing the possibility of altering the surface chemistry by the addition of ionic liquids. Remarkably, a strong interaction between the ILs and WCU4 surface was perceived, acting the former as a reducing agent. Indeed, this interaction transforms the WO_3_ in tungsten suboxides, which are known to present good catalytic activity for the hydrogen evolution reaction.

Finally, it was observed by DEMS an overpotential value of 45 mV for the WCU- EPy toward HER in alkaline media. This implies a shift of 55 mV in the onset potential for the HER toward positive potentials when EPyPF_6_ is added to WCU4. It is also proposed that DEMS provides not only accurate onset potential for the HER but also precise determination of kinetics and mechanistic parameters. Tafel slope values acquired with ionic currents reveal that the RDS changes from Heyrovsky-Volmer to Heyrovsky when the suboxide structures are present on the surface of the catalysts. This implies faster kinetics in the reduction of water molecules in the tungsten suboxide structures at high pH values. Thus, modification of carbide powder surfaces with ionic liquids is proposed as a fast, cheap, and effective route to enhance the performance of tungsten carbide surfaces toward HER in alkaline media.

## Supporting Information Available

SEM, XPS, and DEMS analyses; DEMS configuration and delay time; additional DEMS measurement of WCU2-EPy; determination of Tafel slopes from DEMS ionic currents; capacitance and impedance towards alkaline HER; and references (PDF)
