# Supplementary material for: Highly Active W2C-Based Composites for the HER in Alkaline Solution: the Role of Surface Oxide Species
Source: ACS Appl Mater Interfaces. 2024 Apr 22;16(17):21877–84. doi: 10.1021/acsami.4c01612 (PMC11071040; doi:10.1021/acsami.4c01612)
Supplement: Supplementary file 1 — am4c01612_si_001.pdf [file am4c01612_si_001.pdf]

Supporting Information

Highly active W_2C-based composites for the HER in alkaline solution. The role of surface oxide species

S. Díaz-Coello[a,b], D. Winkler[b], C. Griesser[b], T. Moser[b], J.L. Rodríguez[a], J. Kunze-Liebhäuser[b], G. García[a], E. Pastor[a,*]

[a] Institute of Materials and Nanotechnology, Department of Chemistry, University of La Laguna, PO Box 456, 38200, La Laguna, Santa Cruz de Tenerife, Spain.

[b] Department of Physical Chemistry, University of Innsbruck, Innrain 52c, Innsbruck, Austria.

*Corresponding author:

E-mail: epastor@ull.edu.es (E. Pastor); Phone: +34 922318071

Table of Contents

1. Scanning Electron Microscopy (SEM) 1

2. X-ray photoelectron spectroscopy (XPS) 2

3. Electrochemistry and Differential Electrochemical Mass Spectrometry (DEMS) 5

3.1. DEMS configuration and delay time 5

3.2. Additional DEMS measurement of WCU2-EPy 7

3.3. Determination of Tafel slopes from DEMS ionic currents 7

3.3. Capacitance and impedance towards alkaline HER 9

4. References 10

1. Scanning Electron Microscopy (SEM)

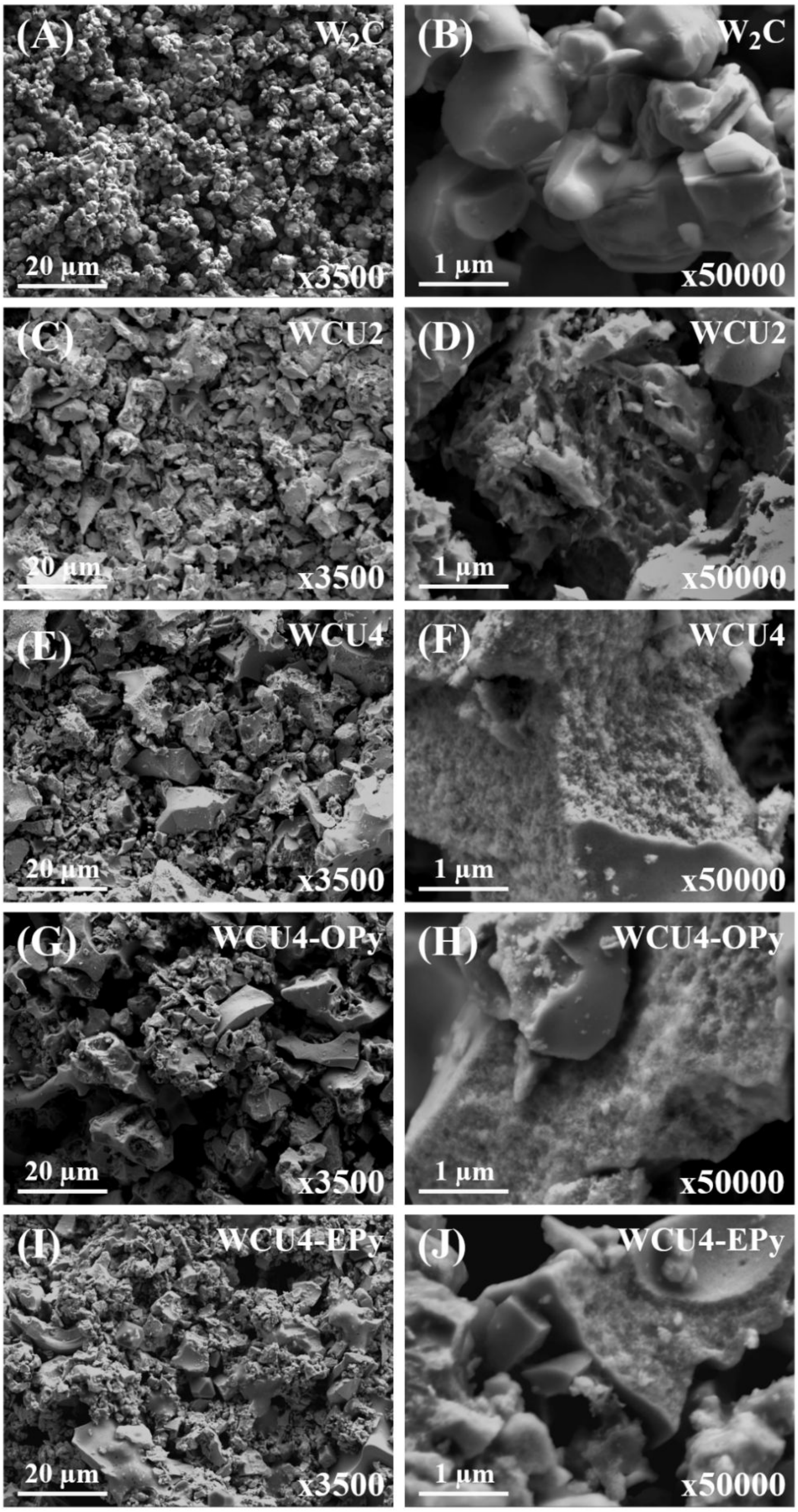

Figure S1. SEM images obtained for commercial W_2C **(A, B)**, WCU2 **(C, D)**, WCU4 **(E, F)**, WCU4-OPy **(G, H)** and WCU4-EPy **(I, J)**. Images have been recorded using a secondary electron detector at x3500 (left) and x50000 (right) magnification for each sample.

2. X-ray photoelectron spectroscopy (XPS)

Here, the complete survey and all the regions of interest of the XPS spectra can be consulted for all materials studied in this work (Figures S1-S5 and Table S1). Apart from the features mentioned in the main article, it is worthy to further explain the signals related to the W_2C/IL composite materials. In these samples, the spectra at the C 1s region reveal the signals related to the carbon atoms of the N-alkylpyridinium cation (see Figures S4 and S5). According to the literature, the signals have been identified as $C_{aliphatic}$ for the carbon of the side chain, C_{inter} for the three carbon atoms placed opposite to the nitrogen, and C_{hetero} for the three carbon atoms directly bonded to the nitrogen [1]. Regarding the interaction of the ionic liquids with the surface oxides, the length of the carbon sidechain of the cation appears to affect the amount of surface oxides reduced. However, no evidence of other carbon-based compounds produced by the reduction process have been detected by XPS. On the other hand, the O 1s region reveals a signal ca. 534.5 eV which has been associated with the formation of P-O bonds [2]. This signal is probably caused by the interaction between the PF_6^- counterion of the ionic liquid and the surface oxides of the carbide. Interestingly, the P-O signal increases as the percentage of WO_3 decreases and so the PF_6^- anion is most likely responsible for the reduction of the surface.

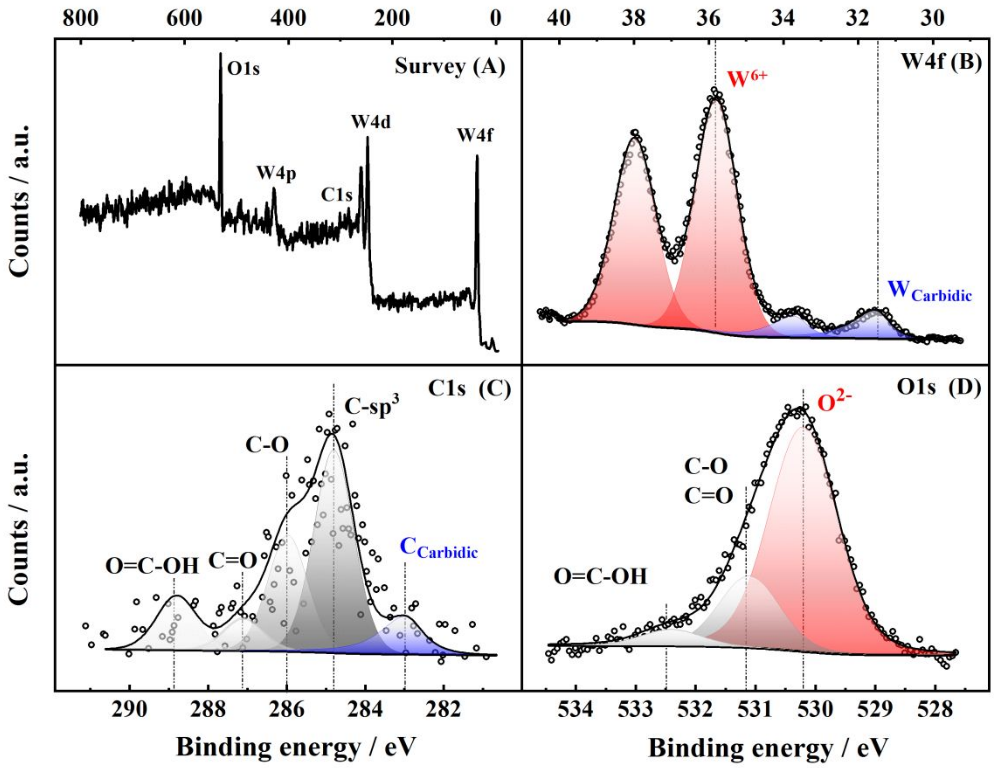

Figure S2. XPS survey (top left), high-resolution scans for W 4f (top right), C 1s (bottom left) and O 1s (bottom right) regions recorded for commercial W_2C.

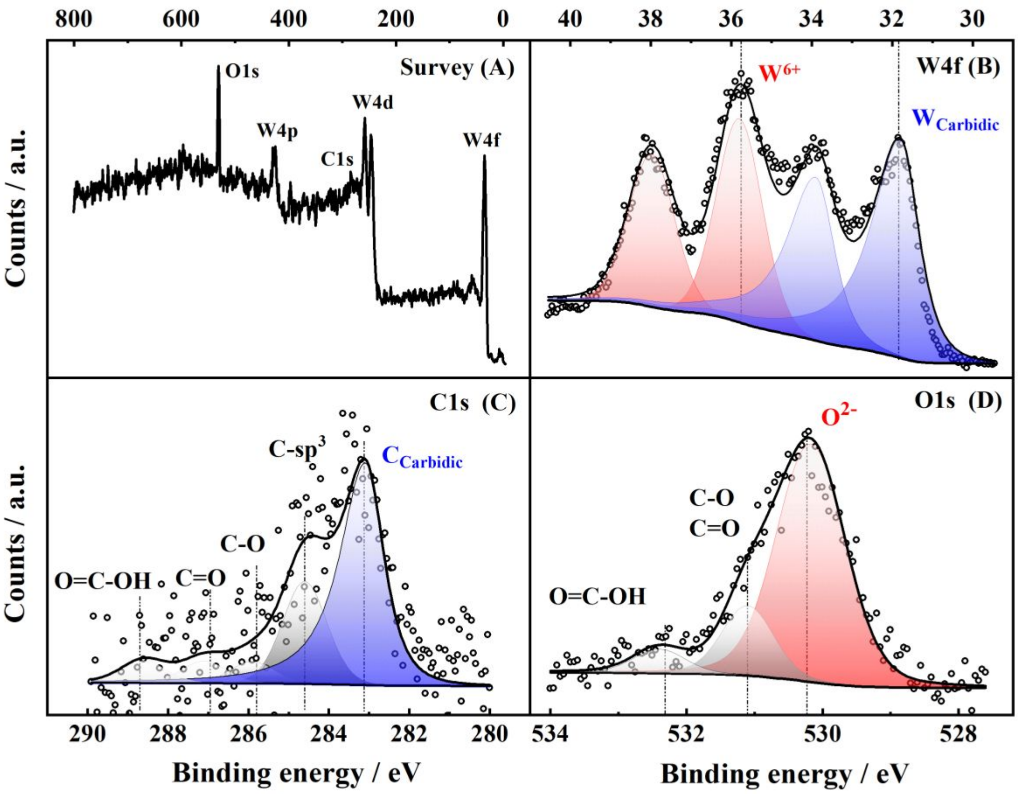

Figure S3. XPS survey (top left), high-resolution scans for W 4f (top right), C 1s (bottom left) and O 1s (bottom right) regions recorded for WCU2.

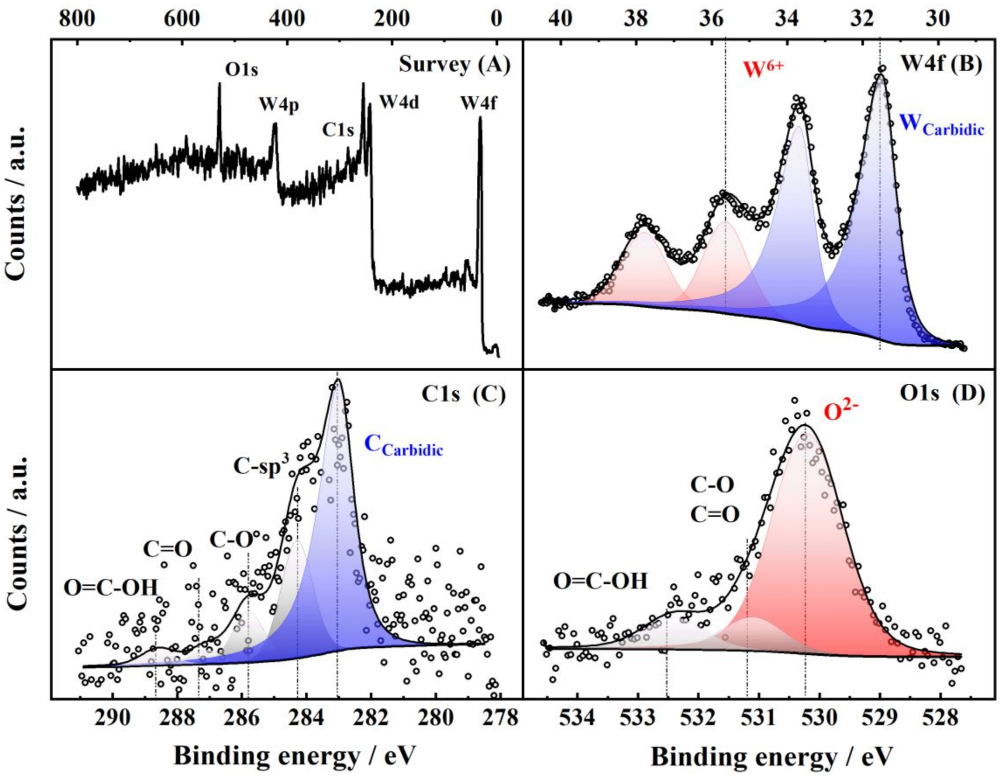

Figure S4. XPS survey (top left), high-resolution scans for W 4f (top right), C 1s (bottom left) and O 1s (bottom right) regions recorded for WCU4.

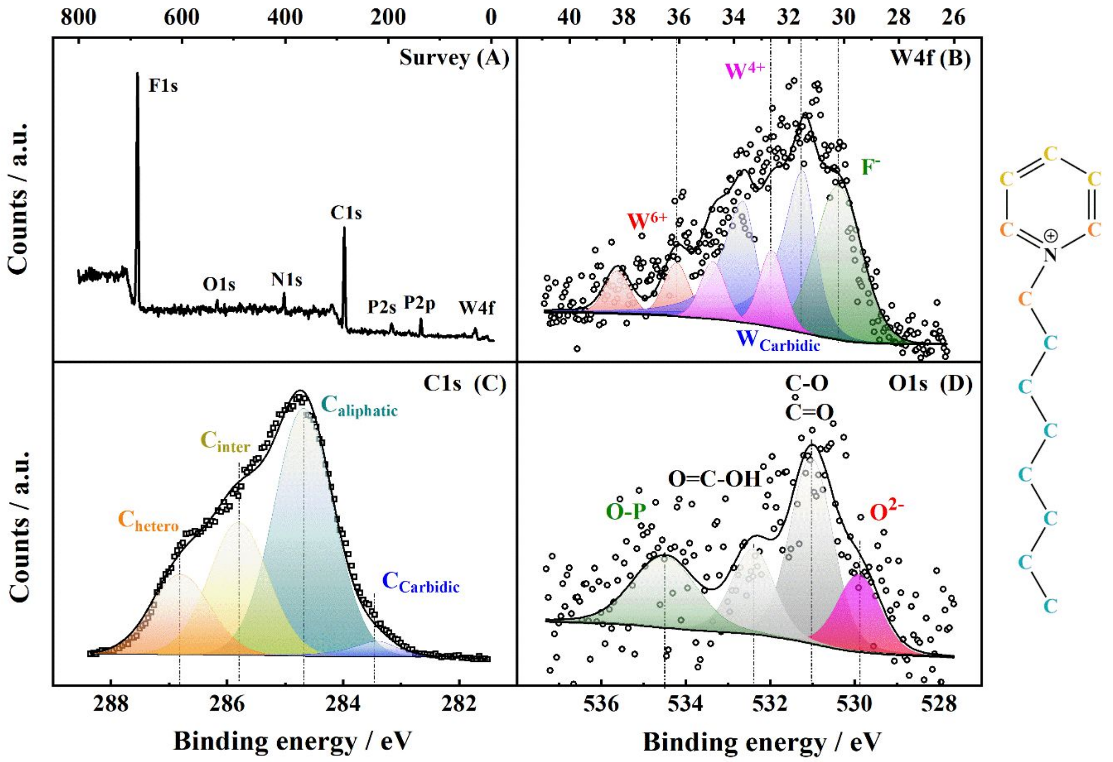

Figure S5. XPS survey (top left), high-resolution scans for W 4f (top right), C 1s (bottom left) and O 1s (bottom right) regions recorded for WCU4-OPy. At the right margin the octylpyridinium cation is shown.

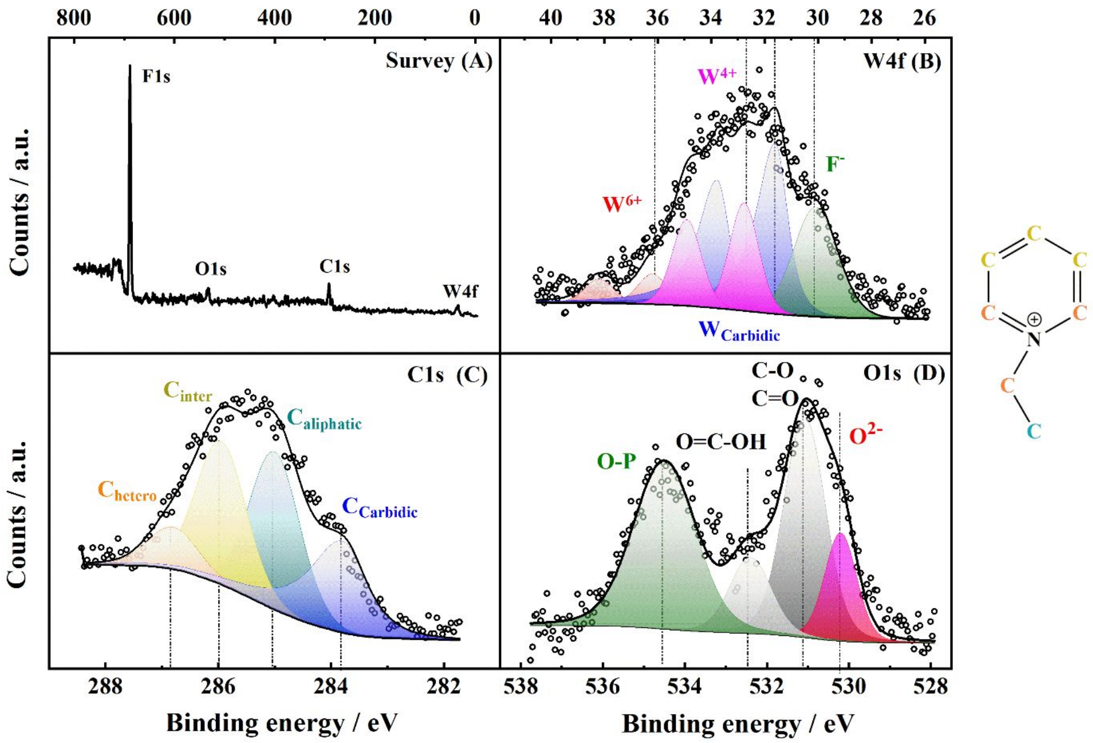

Figure S6. XPS survey (top left), high-resolution scans for W 4f (top right), C 1s (bottom left) and O 1s (bottom right) regions recorded for WCU4-EPy. At the right margin the ethylpyridinium cation is shown.

Table S1. XPS fitting parameters for commercial W_2C, synthesized carbides (WCU2 and WCU4) and composite materials (WCU4-OPy and WCU4-EPy). The % column indicates the percentage of tungsten signals.

Catalyst	W 4f	BE (eV)	FWHM (eV)	%	$(W^{2+} + W^{4+}) / W^{6+}$ Surface ratio
W_2C	W^{2+}	31.5	0.9	6.1	0.06
	W^{4+}	-	-	-	
	W^{6+}	35.8	1.3	93.9	
WCU2	W^{2+}	31.7	1.0	46.5	0.87
	W^{4+}	-	-	-	
	W^{6+}	35.8	1.3	53.5	
WCU4	W^{2+}	31.5	0.9	55.6	1.25
	W^{4+}	-	-	-	
	W^{6+}	35.7	1.3	44.4	
WCU4-OPy	W^{2+}	31.5	1.1	67.0	6.29
	W^{4+}	32.6	1.1	19.3	
	W^{6+}	36.1	1.1	13.7	
WCU4-EPy	W^{2+}	31.6	1.1	62.7	11.34
	W^{4+}	32.7	1.2	29.2	
	W^{6+}	36.1	1.2	8.1	

3. Electrochemistry and Differential Electrochemical Mass Spectrometry (DEMS)

3.1. DEMS configuration and delay time

DEMS measurements were carried out in a mass spectrometer (HPR-40, Hiden), using a PEEK-based flow cell. The latter was separated from the mass spectrometer inlet by means of a PTFE membrane (Cobbeter, PS02). The hydrophobic membrane allows volatile and gaseous species generated during the cathodic scan (e.g., hydrogen) to reach the mass spectrometer while it keeps liquid water in the cell. PTFE-based capillaries served as inlet and outlet to flood the cell with the electrolyte. The working electrode consisted of a dispersion of the studied materials dried and immobilized onto a glassy-carbon disk (area = 0.1965 cm^{-2}). The latter was immersed in the cell and adjusted to achieve an electrolyte thin layer configuration between the PTFE membrane by means of a screw. The layer measured approximately 30 μm. Before the

application of the potential scans, the spectrometer valve was opened for 1 h (until the pressure of the chamber was stable; □ $5 \cdot 10^{-6}$ mbar). During the electrochemical swept, the mass spectrometer was set to record the m/z = 2 (H_2^+ fragment) using an ionization cage voltage of 3 V and secondary electron multiplier detector. Further information about the set-up can be found elsewhere [3].

MASSoft software (Hiden) was used to control the mass spectrometer, recording at the same time the m/z = 2 ionic current and the potentials applied by the potentiostat/galvanostat. To elaborate the mass linear sweep voltammetry (MSLSV) graph, the m/z = 2 ionic current was graph versus the applied potential and depicted along with the respective j *vs*. E plot.

There is an inherent limitation of DEMS technique normally referred as delay time. This is defined as the time that takes to the generated gaseous/volatile species to travel from the electrode surface to the spectrometer chamber. It is important to carry the measurements at slow electrochemical scan rates (e.g., $1\ mV \cdot s^{-1}$) to avoid excessively high delay times, and thus the loss of the electrochemical online experiment. The delay time can be calculated as (i) the time difference between the potential sweep and the detected ionic current during a cyclic voltammetry; or (ii) the response time of the ionic current during a chronoamperometry. In the case of the experiments recorded in this work, Figure S6A shows the applied potential as a function of the time along with the detected m/z = 2 ionic currents detected during the DEMS measurement of the W_2C sample. The time difference between the potential swept and the ionic currents is depicted by the dot lines (Figure S6B shows a magnification of the region). Thus, it is established that the DEMS configuration used in this work provides a delay time of approximately 3 s and online detection of hydrogen can be assumed.

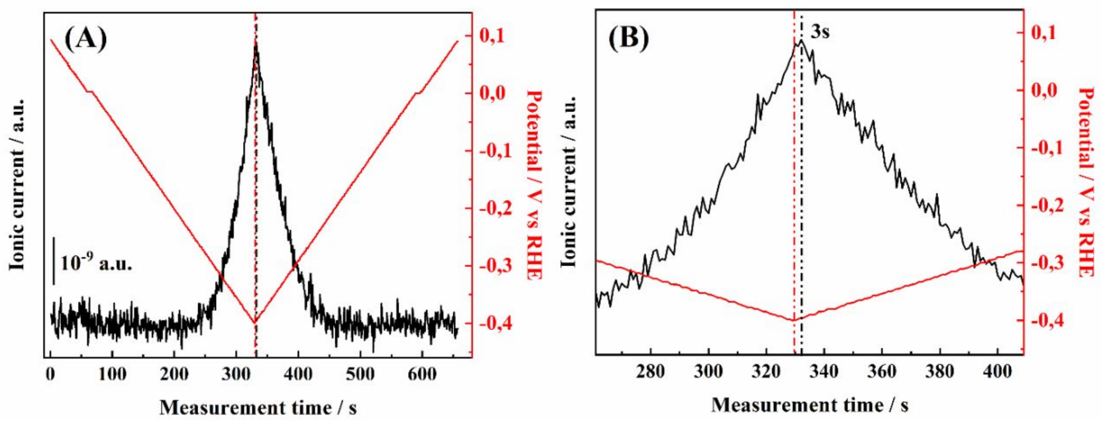

Figure S7. (A) m/z = 2 ionic currents and applied potentials depicted versus time. Both signals were recorded during the DEMS experiments in 0.1 M NaOH at $1\ mV \cdot s^{-1}$ for the commercial W_2C. **(B)** Magnification of the region between 280 and 400 s. In both panels, the black dotted

line indicates the maximum signal, and the red dotted line indicates the change at the potential swept direction.

3.2. Additional DEMS measurement of WCU2-EPy

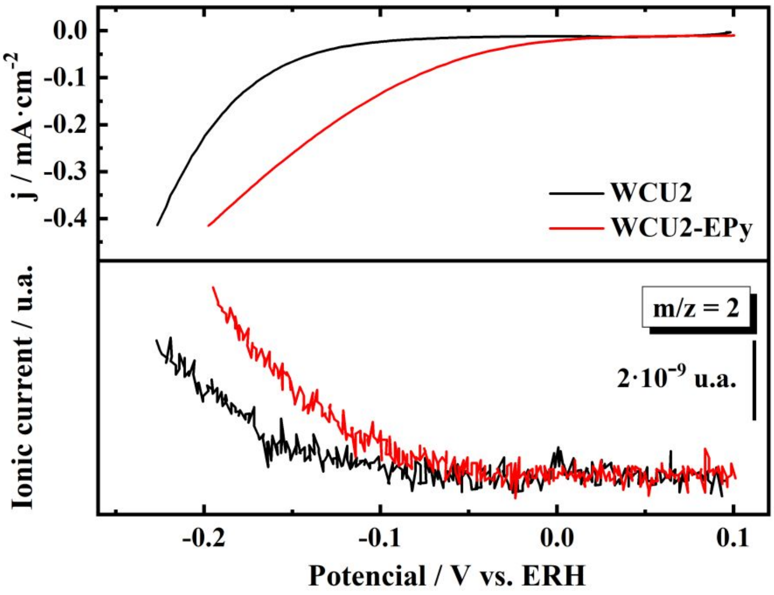

Figure S8. (A) LSVs (top panel) with corresponding MSLSVs for m/z = 2 (bottom panel) signals obtained in the DEMS experiments performed with WCU2 and WCU2-EPy catalysts. The LSVs were recorded starting at 0.1 V_{RHE} and going towards negative potentials at 1 mVs^{-1} in 0.1 M NaOH (IUPAC convention) using a GC rod as counter electrode, a KCl_{sat} Ag/AgCl reference electrode and a GC disk as substrate for the working electrode material. The procedure for the modification of the substrate can be found in the experimental section.

3.3. Determination of Tafel slopes from DEMS ionic currents

We propose that it is possible to obtain reliable kinetic information of the process from the ionic currents recorded during a DEMS experiment. This is based on the works first reported by Wolter and Heitbaum [4], and then by Baltruschat [5] where they proved that during an online DEMS experiment the ionic currents are proportional to the faradaic currents recorded at the working electrode according to Equation. S1.

$$i_i = \frac{K_0\,N}{z\,F} i_F \qquad \text{S1}$$

Where i_I represents the ionic current at the mass spectrometer, i_F is the faradaic current, K_0 is the instrumental constant, z is the number of electrons and F the Faraday constant. This mathematical relation has been widely corroborated and used in different systems. Some

examples are the calculation of the faradaic efficiency to CO_2 during ethanol oxidation on Pt-based materials [6] or the same reported for O_2 and H_2 generation as well as for isopropanol oxidation to acetone [7].

On the other hand, Tafel slopes can be described as a numerical value indicating how fast the faradaic current increases for each potential step. Taking into consideration that both faradaic and ionic currents are proportional according to equation S1, then solving the Tafel slopes from ionic currents indicates the kinetics of the HER without by-side reactions. To prove this concept, Figure S7A and Figure S7B depict the Tafel slopes obtained from both faradaic and ionic currents, respectively, for the materials studied in this work. First, all the Tafel slopes obtained from ionic currents are always smaller than their respective faradaic-derived. This is caused by the contribution of different by-side reactions to the total faradaic current. In the case of the materials reported in this work, the surface oxides are being reduced in the same region as the hydrogen evolution reaction. The effect can be clearly seen as the faradaic-derived Tafel slope of the W_2C (93 % of surface WO_3) have a value of 295 $mV\cdot dec^{-1}$ while the WCU2 and WCU4 (44 – 53 % of surface WO_3) present values around 90 $mV\cdot dec^{-1}$. Thus, the effect of the surface oxides is clearly playing a role on the determination of the rate determining step (i.e. faradaic-derived Tafel slopes are higher as the surface oxide increase). When the Tafel slopes are calculated with the m/z = 2 ionic currents from the DEMS experiment, all values are restricted to those theoretically accepted and the slope for the commercial W_2C changes from 295 to 68 $mV\cdot dec^{-1}$. This value is consistent with those obtained with the same method for the synthesized sample, which is expectable as all of them present the same structures as electroactive phase for the HER (hexagonal W_2C). Therefore, DEMS is proposed as a suitable technique for the determination of kinetic parameters.

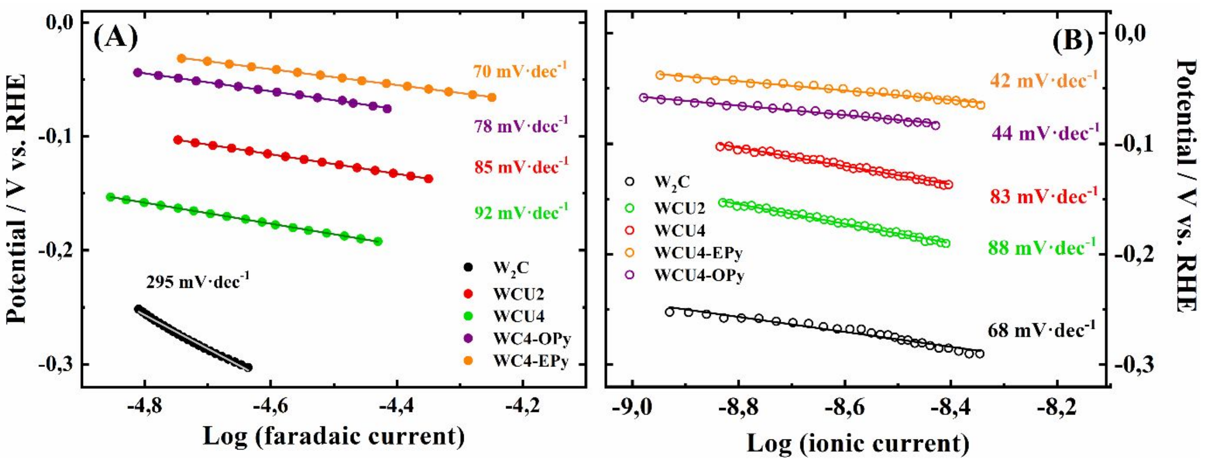

Figure S9. Faradaic-derived **(A)** and ionic-derived **(B)** Tafel slopes calculated from the DEMS experiment depicted in Figure 3 of the main article.

3.3. Capacitance and impedance towards alkaline HER

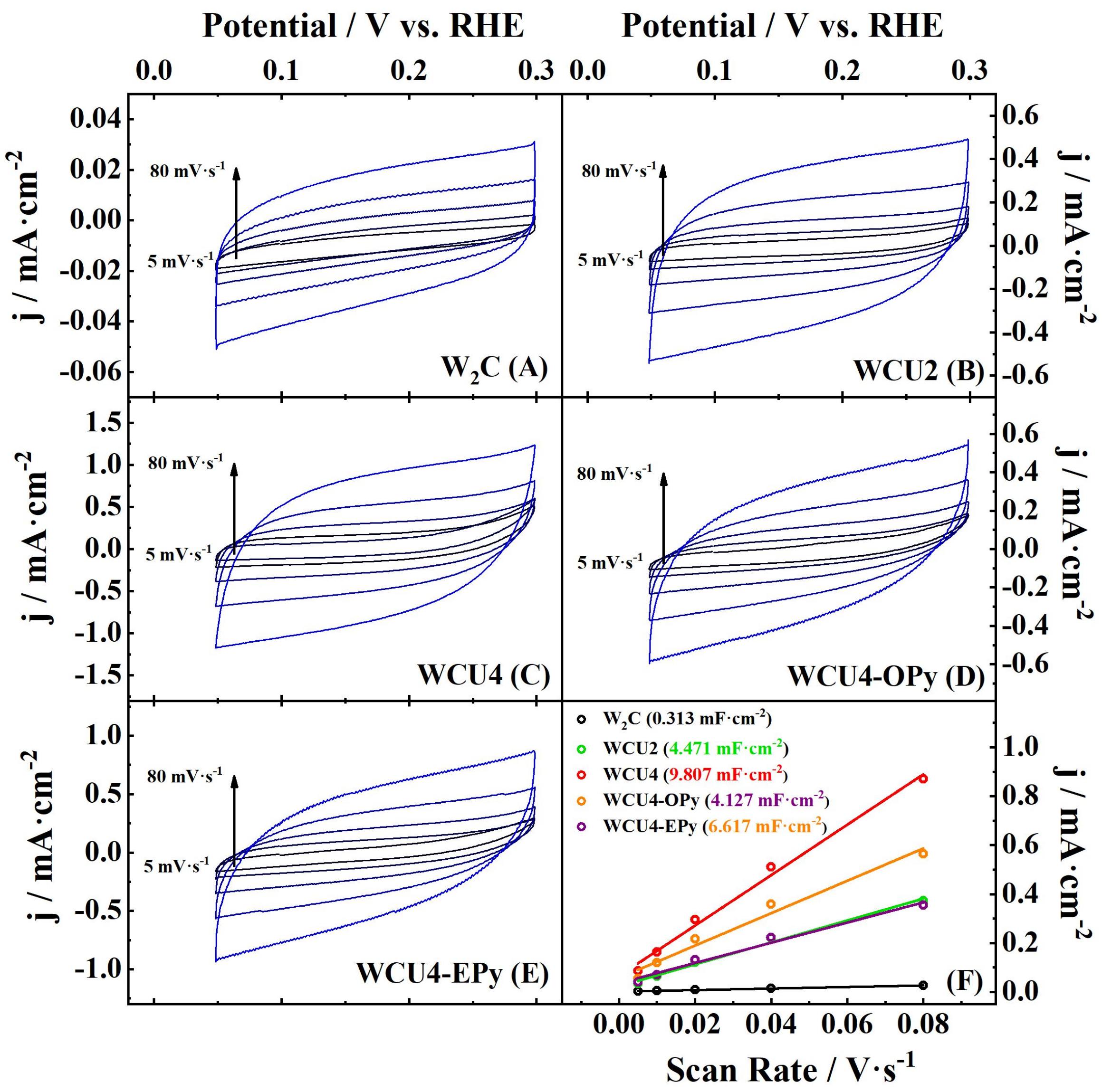

Figure S10. CVs experiments performed with **(A)** W_2C, **(B)** WCU2, **(C)** WCU4, **(D)** WCU4-OPy and **(E)** WCU4-EPy catalysts recorded at different scan rates (5, 10, 20, 40, and 80 $mV \cdot s^{-1}$) between 0.05 and 0.3 V_{RHE} in 0.1 M NaOH. **(F)** Capacitive currents vs. sweep scan rate depicted for every material for the evaluation of the capacitance. All results have been obtained by using GC rod as counter electrode, a KCl_{sat} Ag/AgCl reference electrode and a GC disk as substrate for the working electrode material. The procedure for the modification of the substrate can be found in the experimental section of the main article. All results are depicted using the IUPAC convention.

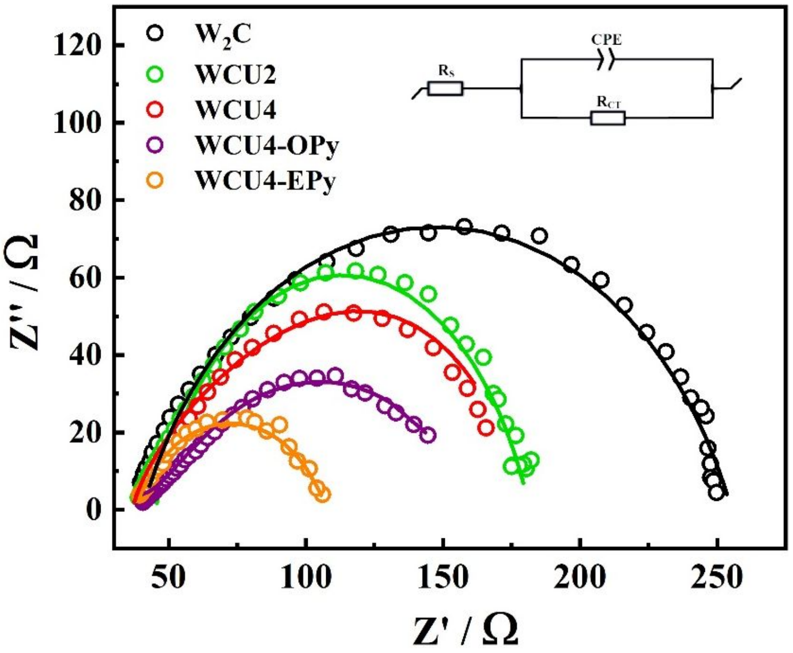

Figure S11. Nyquist plot semicircles of commercial W_2C, WCU2, WCU4, WCU4-EPy and WCU4-OPy recorded at −0.25, -0.18, -0,15, -0,09 and -0,05 V vs. RHE, respectively, in 0.1 M NaOH. The data have been fitted to the equivalent circuit shown in the inset. R_S represents the electrical resistance of the electrolyte solution; CPE is the constant phase element, and R_{CT} is the electrical resistance of the charge transfer during the reaction.

Table S2. Summary of parameters solved by EIS experiments.

Sample	R_S / Ω	Q / mF	a	R_{CT} / Ω
W_2C	40.1	0.0748	0,763	285.8
WCU2	40.4	0.0334	0.760	143.9
WCU4	39.3	0.0921	0,752	140.0
WCU4-OPy	40.6	1.32	0.569	133.4
WCU4-EPy	41.2	1.08	0.772	70.2

R_S: Electrical resistance of the electrolyte
Q: Capacitance of the constant phase element
a: Correction factor of the constant phase element
R_{CT}: Electrical resistance of the charge transfer during faradaic reaction

4. References

[1] Men, S.; Mitchell, D. S.; Lovelock, K. R. J.; Licence, P. X-ray Photoelectron Spectroscopy of Pyridinium-Based Ionic Liquids: Comparison to Imidazolium- and

Pyrrolidinium-Based Analogues. *ChemPhysChem* **2015**, 16 (10), 2211–2218. DOI: 10.1002/cphc.201500227.

[2] Wang, Y.; Lan, Z.; Xu, F.; Zhao, L.; Xu, J.; Chen, J.; Liu, R.; Du, Z.; Du, H.; Ma, Z. Exploration of Phosphorus Oxide in Heavily Phosphorus-Doped Polysilicon Films of Tunneling Oxide Passivation Contact Solar Cells. *ACS Appl. Electron. Mater.* **2022**, 4 (8), 3947–3954. DOI: 10.1021/acsaelm.2c00604.

[3] Winkler, D.; Leitner, M.; Auer, A.; Kunze-Liebhäuser, J. The Relevance of the Interfacial Water Reactivity for Electrochemical CO Reduction on Copper Single Crystals. *ACS Catal*. **2024**, 14, 2, 1098–1106. doi: 10.1021/acscatal.3c02700

[4] Wolter, O.; Heitbaum, J.; Differential Electrochemical Mass Spectrometry (DEMS) – A New Method for the Study of Electrode Processes. *Ber. Bunsenges. Phys. Chem.* **1984**, 88, 1, 2–6. doi: 10.1002/bbpc.19840880103.

[5] Baltruschat, H. Differential Electrochemical Mass Spectrometry. *J. Am. Soc. Mass Spectrom.* **2004**, 15, 12, 1693–1706. doi: 10.1016/j.jasms.2004.09.011.

[6] Rizo, R.; Sebastián, D.; Rodríguez, J.L.; Lázaro, M.J.; Pastor, E. Influence of the Nature of the Carbon Support on the Activity of Pt/C Catalysts for Ethanol and Carbon Monoxide Oxidation. *J. Catal*. **2017**, 348, 22–28. doi: 10.1016/j.jcat.2017.02.007.

[7] Bondue, C.J.; Koper, M.T.M.; Tschulik, K. A Versatile and Easy Method to Calibrate a Two-Compartment Flow Cell for Differential Electrochemical Mass Spectrometry Measurements. *ACS Measurement Science Au* 2023, 3, 4, 277 – 286. doi: 10.1021/acsmeasuresciau.3c00009.
